# PLAAT1 Exhibits Phosphatidylcholine:Monolysocardiolipin Transacylase Activity

**DOI:** 10.3390/ijms23126714

**Published:** 2022-06-16

**Authors:** Ryan M. Bradley, Ashkan Hashemi, Juan J. Aristizabal-Henao, Ken D. Stark, Robin E. Duncan

**Affiliations:** Department of Kinesiology and Health Sciences, Faculty of Health, University of Waterloo, 200 University Ave W, BMH1044, Waterloo, ON N2L 3G1, Canada; ryanbradley94@gmail.com (R.M.B.); a4hashem@uwaterloo.ca (A.H.); juan.henao@berghealth.com (J.J.A.-H.); kstark@uwaterloo.ca (K.D.S.)

**Keywords:** cardiolipin, monolysocardiolipin, remodeling enzyme, phospholipase A, acyltransferase 1 (PLAAT1)

## Abstract

Tissue-specific cardiolipin fatty acyl profiles are achieved by remodeling of de novo synthesized cardiolipin, and four remodeling enzymes have thus far been identified. We studied the enzyme phospholipase A and acyltransferase 1 (PLAAT1), and we report the discovery that it has phosphatidylcholine (PC):monolysocardiolipin (MLCL) transacylase activity. Subcellular localization was analyzed by differential centrifugation and immunoblotting. Total levels of major phospholipids, and the fatty acyl profile of cardiolipin, were analyzed in HEK293 cells expressing murine PLAAT1 using gas chromatography. Apparent enzyme kinetics of affinity-purified PLAAT1 were calculated using radiochemical enzyme assays. This enzyme was found to localize predominantly to the endoplasmic reticulum (ER) but was detected at low levels in the mitochondria-associated ER matrix. Cells expressing PLAAT1 had higher levels of total cardiolipin, but not other phospholipids, and it was primarily enriched in the saturated fatty acids myristate, palmitate, and stearate, with quantitatively smaller increases in the n-3 polyunsaturated fatty acids linolenate, eicosatrienoate, and eicosapentanoate and the monounsaturated fatty acid erucate. Affinity-purified PLAAT1 did not catalyze the transacylation of MLCL using 1-palmitoyl-2-[^14^C]-linoleoyl-PC as an acyl donor. However, PLAAT1 had an apparent V_max_ of 1.61 μmol/min/mg protein and Km of 126 μM using [9,10-^3^H]-distearoyl-PC as an acyl donor, and 0.61 μmol/min/mg protein and Km of 16 μM using [9,10-^3^H]-dioleoyl-PC. PLAAT1 is therefore a novel PC:MLCL transacylase.

## 1. Introduction

Cardiolipin is a unique dimeric phospholipid required for proper cristae architecture within the mitochondria [1]. Although 18-carbon fatty acids predominate in cardiolipin in most tissues, the fatty acyl composition of this lipid varies between organs, reflecting the diversity of functional considerations of each tissue, including the need to balance energetic requirements with the inherent toxicity of oxidative metabolism [2]. For example, the cardiolipin profile of the brain tends to be more highly enriched in saturated and monounsaturated (i.e., stearoyl- and oleoyl-rich) fatty acids that are less susceptible to oxidative damage, compared to cardiac tissue that has a higher energetic demand, but also a greater capacity for repair, and is predominantly (i.e., >80%) tetra-linoleoyl [2].

Cardiolipin is produced de novo in the Kennedy pathway. At initial synthesis, it is considered “immature” or “nascent”, and it must subsequently be remodeled to contain a fatty acyl chain profile that is functionally appropriate for the particular tissue [3]. This chemical specificity is achieved through a process that utilizes phospholipase, acyltransferase, and transacylase enzymes [4,5,6]. Remodeling of “immature” cardiolipin occurs in Lands’ pathway and begins with phospholipases cleaving fatty acyl chains from either one or both glycerol backbones, resulting in the production of monolysocardiolipin (MLCL) or dilysocardiolipin (DLCL) [7]. Mature, fully acylated cardiolipin is then reconstituted through the action of either an acyl-CoA-dependent acyltransferase, which utilizes fatty acyl-CoAs as donor substrates, or transacylases, which catalyze the transfer of a fatty acyl chain from a phospholipid such as phosphatidylcholine (PC) to the lysocardiolipin molecule, generating a new lysophospholipid from the acyl donor [8]. Differences in the enzymes involved in remodeling, and the preferential substrates utilized, result in tissue-specific cardiolipin fatty acyl profiles [3]. Maintenance of these profiles is critical to health, since alterations are associated with a variety of diseases, including diabetes [9,10,11], inflammatory and autoimmune disorders [12,13], non-alcoholic fatty liver disease [14], cardiomyopathies [15] and cardiovascular disease [16], several types of cancer [17], and neurodegenerative diseases such as Alzheimer’s [18] and Parkinson’s diseases [19].

Four cardiolipin remodeling enzymes have been identified. The first was a mitochondrial linoleoyl transacylase called tafazzin that was discovered in 1996 [20]. This enzyme is expressed at the highest levels in cardiac muscle, where it predominantly uses linoleate residues on phosphatidylcholine to remodel MLCL [21]. A series of different mutations in tafazzin have been identified in humans, and these adversely affect cardiolipin remodeling, resulting in Barth syndrome which is characterized by skeletal and cardiac myopathies and neutropenia [22]. The Hatch Laboratory cloned and characterized MLCL acyltransferase (AT)-1 in 1999 and reported that it is highly expressed in rodent cardiac tissue [23]. This enzyme preferentially uses linoleoyl-CoA and oleoyl-CoA as acyl donors, and MLCL as the acyl acceptor, and is lacking in activity with other lysophospholipid substrates including DLCL [23]. In 2009, this same laboratory identified the human homolog of MLCL AT-1 as the α-subunit of human trifunctional protein (αTFP) [5]. ALCAT1 was identified in 2004 as an acyl-CoA:lysocardiolipin acyltransferase that can use both MLCL and DLCL as acyl acceptors [4].

Here, we report the discovery that a fifth enzyme, phospholipase A and acyltransferase 1 (PLAAT1), also catalyzes the transacylation of monolysocardiolipin (MLCL) using phosphatidylcholine (PC) as an acyl donor. This enzyme, also known as A-C1, Harvey-Ras-like tumor suppressor (HRASLS), or HRASLS1, is a member of a homologous group of proteins. All known PLAAT enzymes possess lipid enzymatic activities, including phospholipase A1/2 (PLA) and O- and N-transacylase activities [24]. However, none have yet been described as having functions in cardiolipin synthesis or remodeling. In this work, we present evidence that PLAAT1 has PC:MLCL transacylase activity, and therefore a direct role in cardiolipin metabolism. Potential implications of this discovery for understanding health and disease are discussed, including possible implications for diabetes.

## 2. Results

### 2.1. PLAAT1 Expression and Localization

**Figure 1:**
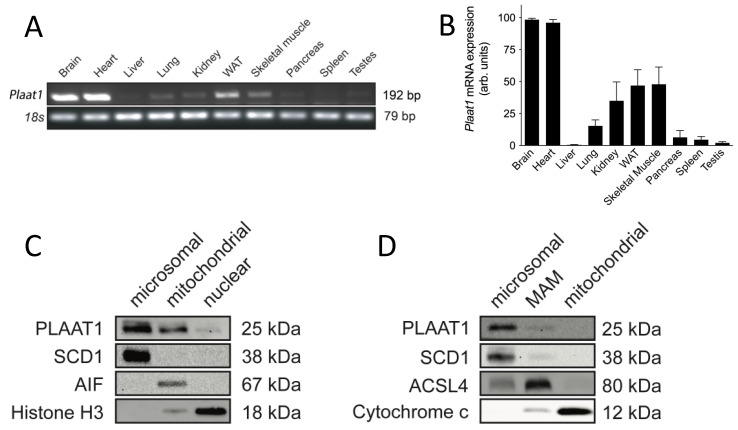
**PLAAT1 expression and subcellular localization.***Plaat1* gene expression was detected at highest levels in brain, heart, white adipose tissue (WAT), and skeletal muscle, but was also visualized (**A**) and found by density analysis of bands (**B**) to be present in other tissues at lower levels. Data are means ± S.E.M., *n* = 3–6. Subcellular localization of PLAAT1 was investigated by immunoblotting microsomal, mitochondrial, and nuclear fractions produced by differential centrifugation of whole mouse brains for PLAAT1, or for markers of fractional purity including SCD1 as a marker of the endoplasmic reticulum (ER), AIF as a mitochondria-specific marker, and histone H3 as a nuclear marker (**C**). Alternately, mouse brains were separated to derive ER (microsomal), mitochondria-associated ER matrix (MAM), and mitochondrial fractions for detection of PLAAT1 or markers of fractional purity (i.e., SCD1 (ER), ACSL4 (MAM), and cytochrome c (mitochondria)) (*n* = 3) (**D**).

Mouse tissue distribution of *Plaat1* mRNA (transcript variant 1) was analyzed by semi-quantitative RT-PCR. In agreement with Hussein et al. [25], it was found to be expressed in a variety of tissues but was detected in the greatest abundance in the mouse brain, heart, and skeletal muscle (Figure 1A,B and Figure S1). An additional analysis from whole blood did not result in amplification of the expected target transcript, suggesting very low abundance, in agreement with the work of others (data not shown) [25,26]. Whole-brain homogenates were fractionated by differential centrifugation and immunoblotted to detect endogenous PLAAT1. Although this protein is predicted to be ~18.4 kDa in mass, immunodetectable PLAAT1 was visualized between 20 kDa and 25 kDa on immunoblots (Figure 1C,D, Figures S2 and S3), in agreement with the findings of others [25]. This enzyme was strongly detected in the microsomal fraction, indicating localization to the endoplasmic reticulum (ER). In addition, a faint band was also detected in the mitochondrial fraction (Figure 1C). Fractional purity was analyzed by immunoblotting for the mitochondrial protein cytochrome C, the nuclear protein histone H3, and the endoplasmic reticular protein stearoyl-CoA desaturase 1 (SCD1) (Figure 1C). Although fractional purity was high, detection of PLAAT1 in the mitochondrial fraction could have been due to minor cross-contamination from other organelles. Alternately, PLAAT1 may have been present in a fraction that would potentially co-fractionate with the mitochondria and the ER, such as the mitochondria-associated endoplasmic reticulum membrane (MAM). Mitochondrial isolates were therefore further processed to separate the purified mitochondrial fraction from a purified microsomal fraction and a MAM fraction. Fractional purity was assessed by immunoblot analysis of the MAM-specific protein acyl-CoA synthase long-chain family member 4 (ACSL4), as well as mitochondrial cytochrome C and microsomal SCD1. Immunoblot analysis of isolated subfractions showed that endogenous PLAAT1 was detected again at the highest levels in the microsomal fraction, although a slight band also appeared in the MAM fraction (Figure 1D). Endogenous PLAAT1 was not visible in the mitochondrial fraction once the MAM was separated out (Figure 1D).

### 2.2. Plaat1 Expression Increases Cellular Cardiolipin Content

**Figure 2:**
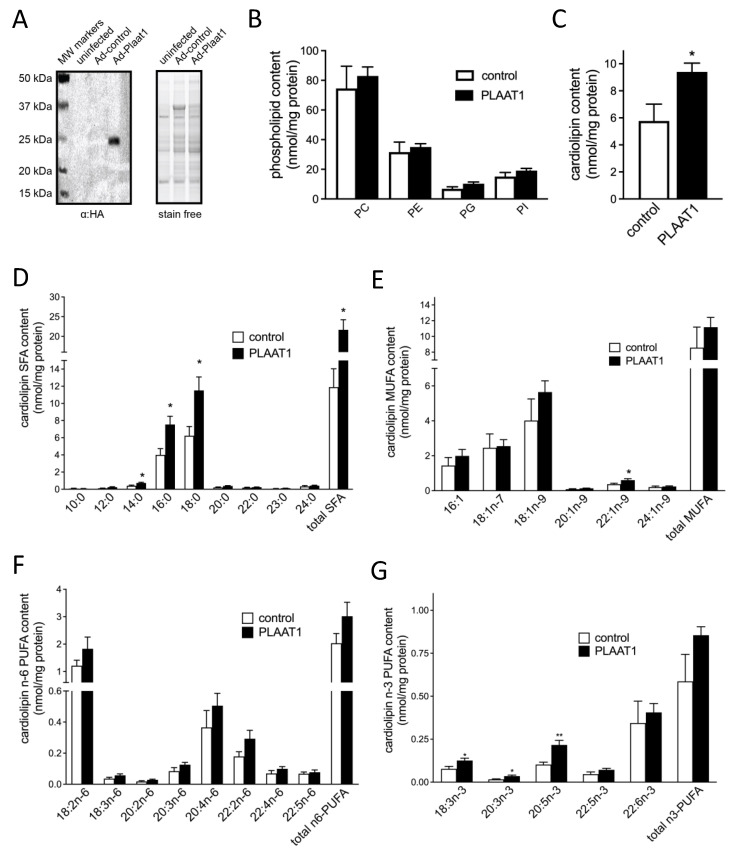
**PLAAT1 expression in HEK-293 cells increases cardiolipin content.** A representative immunoblot of HA-tagged PLAAT1 in HEK-293 cells is shown (left panel) with total protein loading imaged under UV light using a stain-free gel (right panel) (**A**). In HEK-293 cells expressing PLAAT1, or in control cells, the contents of major phospholipid species (**B**) and cardiolipin (**C**) were determined using gas chromatography. Analysis of the fatty acyl composition of cardiolipin is reported as individual and total saturated fatty acids (SFAs) (**D**), monounsaturated fatty acids (MUFAs) (**E**), n-6 polyunsaturated fatty acids (PUFAs) (**F**), and n-3 PUFAs (**G**). Data are means ± S.E.M. (*n* = 8–9). * *p* < 0.05, ** *p* < 0.01.

*Plaat1* encoded by murine transcript variant 1 was expressed in HEK-293 cells (Figure 2A), and major phospholipid categories were isolated for analysis by gas chromatography (Figure 2B,C). Relative to control cells, cells expressing *Plaat1* had 62% more total cardiolipin (5.77 ± 1.24 nmol cardiolipin/mg protein versus 9.40 ± 0.66 nmol cardiolipin/mg protein, respectively, *p* = 0.018) (Figure 2C), while total contents of PC, phosphatidylethanolamine (PE), phosphatidylglycerol (PG), and phosphatidylinositol (PI) were not significantly elevated (Figure 2B). This elevation in total cardiolipin content was associated with specific changes in the cardiolipin fatty acyl profile. Overall, there was a significant increase in the total cardiolipin content of saturated fatty acids (SFAs), while total cardiolipin monounsaturated fatty acids (MUFAs), n-3 polyunsaturated fatty acids (PUFAs), and n-6 PUFAs were not significantly changed (Figure 2D–G). Among individual SFA species, there were significantly higher contents in *Plaat1*-expressing cells compared to controls of myristic acid (0.40 ± 0.08 nmol/mg protein vs. 0.73 ± 0.08 nmol/mg protein, *p* = 0.017), palmitic acid (3.99 ± 0.73 nmol/mg protein vs. 7.54 ± 0.96 nmol/mg protein, *p* = 0.011), and stearic acid (6.24 ± 1.07 nmol/mg protein vs. 11.51 ± 1.57 nmol/mg protein, *p* = 0.016) (Figure 2D). Of the MUFAs analyzed, only erucic acid (22:1n-9) was higher in *Plaat1*-overexpressing cells (Figure 2E), while there were no significant differences in the cardiolipin content of any of the n-6 PUFA species analyzed, including linoleic acid (18:2n-6) (Figure 2F). Among n-3 PUFA species, α-linolenic acid (18:3n-3), eicosatrienoic acid (20:3n-3), and eicosapentanoic acid (20:5n-3) were all increased in cardiolipin from cells expressing *Plaat1* (Figure 2G). Thus, overall, the increased cardiolipin in *Plaat1*-overexpressing HEK-293 cells was associated primarily with a greater content of SFAs and, to some extent, also n-3 PUFA species.

### 2.3. PLAAT1 Is a MLCL:PC Transacylase

The significant increase in cardiolipin content observed in cells expressing PLAAT1 suggested a direct catalytic role for this enzyme in the synthesis of this lipid. Like other members of the PLAAT family, PLAAT1 shares homology with lecithin:retinol acyltransferase (LRAT) which uses PC as an acyl donor [24]. We therefore tested whether affinity-purified PLAAT1 had PC:MLCL transacylase activity, using 100 μM MLCL as an acyl acceptor and increasing concentrations (0–200 μM) of various PC species. It was found that affinity-purified PLAAT1 catalyzed the synthesis of cardiolipin by esterifying [^3^H]18:0 and [^3^H]18:1 fatty acyl chains derived from [9,10-^3^H]-distearoyl-PC and [9,10-^3^H]-dioleoyl-PC, respectively, into MLCL at levels exceeding measures in control reactions, whereas this was not observed for 1-palmitoyl-2-[^14^C]-linoleoyl phosphatidylcholine (Table 1. Thus, kinetic parameters were calculated only for the first two substrates. The calculated V_max_ for PLAAT1 PC:MLCL transacylase activity using [9,10-^3^H]-distearoyl-PC was 1.61 µmol/min/mg protein, and the *K_m_* was 126 µM (Table 1). The calculated V_max_ was lower for [9,10-^3^H]-dioleoyl-PC, at 0.61 µmol/min/mg protein. However, since the *K_m_* was also much lower, at 16 µM, the calculated catalytic efficiency of PLAAT1 with [9,10-^3^H]-dioleoyl-PC was higher than that with [9,10-^3^H]-distearoyl-PC (Table 1).

## 3. Discussion

Cardiolipin synthase (CLS) catalyzes the de novo formation of cardiolipin from phosphatidylglycerol (PG) and CDP-diacylglycerol (CDP-DAG) [27]. CLS, as well as many of the enzymes involved in the synthesis of PG and CDP-DAG, have limited substrate specificity [2]. The fatty acyl profile of nascent cardiolipin therefore typically reflects the general fatty acyl profile of the cell [2]. However, cardiolipin profiles are highly tissue-specific and often exhibit a high degree of compositional similarity between related species, highlighting the importance of cardiolipin form in cell, tissue, or organ function [2]. Achievement of this specificity requires extensive remodeling [2,7,28,29,30]. The present work demonstrates that in addition to the four known enzymes, PLAAT1 also catalyzes the re-esterification of MLCL to form cardiolipin. This constitutes the first identification of a novel cardiolipin remodeling enzyme in over a decade.

PLAAT1 is a member of the PLAAT family of proteins. Five PLAAT/HRASLS enzymes have been identified in humans (PLAAT1-5), while only three are found in mice (PLAAT1, PLAAT3, and PLAAT5) [24]. These enzymes are also known as lecithin:retinol acyltransferase (LRAT)-like proteins, due to their similar sequence homology to this enzyme [8], including a conserved NCEHFV motif in the C-terminal region that is critical for acylation and de-acylation reactions [31]. While all PLAAT family members have previously been reported to have phospholipase A1/2 activity with PC as a substrate, as well as N- and O-transacylase activity using PC as an acyl donor and either PE or lyso-PC, respectively, as acyl acceptors [25,32,33,34,35], activity with MLCL has not previously been reported for this enzyme family.

In comparison to enzymes previously identified in cardiolipin remodeling, this analysis indicates that PLAAT1 is ostensibly most like tafazzin, which also exhibits PC:MLCL transacylase activity, rather than ALCAT1, MLCL AT-1, and αTFP, which all exhibit acyl-CoA-dependent acyltransferase activity [2]. However, our molecular analysis of cardiolipin species in cells expressing PLAAT1 suggested differences in substrate specificity. While tafazzin preferentially esterifies MLCL with linoleate residues from PC [21], the cardiolipin profile of HEK293 cells expressing PLAAT1 was significantly enriched in total and 14-, 16-, and 18-carbon saturated fatty acids and in the n-3 PUFAs linolenate, eicosatrienoate, and eicosanoate. Although ALCAT1 expression also increases the content of n-3 PUFAs in cellular cardiolipin [36], it does this at the expense of saturated and monounsaturated fatty acids, as well as linoleate, which was not observed with PLAAT1 expression.

In vitro analysis of PLAAT1 catalytic activity using affinity-purified enzyme also indicates a novel function that is unique from other known enzymes in cardiolipin remodeling. Unlike MLCL AT-1 or αTFP, which prefer to use linoleoyl-CoA, or tafazzin, which preferentially utilizes linoleate residues from PC, PLAAT1 did not display transacylase activity using linoleate in the *sn-2* position of PC. Conversely, we were able to calculate kinetic parameters for PLAAT1 catalysis when the reacylation of MLCL was analyzed using dioleoyl-PC or distearoyl-PC. While these analyses strongly suggest a substrate preference for 18:0 or 18:1n-9 over 18:2n-6, it is notable that the experimental approach that was used cannot distinguish between substrate selectivity based on positional preference at the *sn-1* or *sn-2* position, or chemical preference for one fatty acyl species type versus another (e.g., stearate or oleate, versus linoleate). PLAAT family enzymes have been shown to have some preference for using fatty acids at the *sn-1* position on the glycerol backbone of PC [24,37]. Thus, it is possible that the lack of transacylase activity observed when 1-palmitoyl-2-[^14^C]-linoleoyl phosphatidylcholine was utilized as an acyl donor resulted from the stereochemical position of the radiolabeled linoleoyl residue in the *sn-2* position, rather than the chemical nature of this species. However, the complete absence of transacylase activity exhibited by PLAAT1 with this substrate makes this notion unlikely, since PLAAT1 does exhibit activity with other PC substrates that indicate an ability to utilize fatty acyl moieties esterified at the *sn-2* position of PC. The apparent enzyme kinetics resulting from this initial evaluation therefore more likely suggest a preference by PLAAT1 for utilization of PC species containing saturated and monounsaturated rather than n-6 polyunsaturated 18-carbon fatty acyl chains, but may also indicate some additional preference for fatty acyl moieties esterified at the *sn-1* position of PC, which together may result in the null activity observed.

Another difference between PLAAT1 and previously identified lipid remodeling enzymes is the subcellular localization. Tafazzin, MLCL AT-1, and αTFP localize to the mitochondria [2]. Although ALCAT1 has been visualized in the endoplasmic reticulum [4], the cardiolipin remodeling activity of this enzyme has been recorded primarily in the MAM and mitochondria [36]. In the current study, PLAAT1 was predominantly detected in the microsomal fraction of whole mouse brains. Although it was also visualized to a much smaller extent in the mitochondrial fraction, further fractionation resulted in a loss of PLAAT1 detection in the mitochondrial fraction, but appearance in the MAM. Detection of PLAAT1 in the endoplasmic reticulum and MAM is not surprising, given the other known roles for this multi-functional enzyme in phospholipid metabolism. It also supports the notion that PLAAT1 may localize to more than one subcellular domain, and in that regard, a recent study has indicated that PLAAT1 can translocate to mitochondria, suggesting PLAAT1 may primarily reside in the endoplasmic reticulum but may be recruited to mitochondria and mitochondria-associated regions when required [38].

The physiological significance of PLAAT1-mediated cardiolipin synthesis remains to be determined and will be the subject of future studies. We, and others [25], have found *Plaat1* to be abundant in mouse brain, heart, and skeletal muscle, although the cardiolipin content of these tissues differs significantly in rodents, with brain enriched primarily in stearate followed by oleate, heart predominantly enriched in linoleate followed by oleate, and skeletal muscle enriched in stearate followed by palmitate [2]. Although dietary fatty acid content can influence mitochondrial cardiolipin composition, the predominance of specific fatty acids in individual tissues tends to be conserved, even in the face of significant changes in diet [2]. This highlights both the importance of enzyme-regulated processes [2] and the importance of tissue-specific profiles for normal tissue function and health. For example, the high abundance of tetra-linoleoyl cardiolipin in the heart is thought to be important in meeting the energy demands of this tissue, while the lower abundance of polyunsaturated fatty acids in brain mitochondria is thought to reflect a balance between generating sufficient energy and limiting the production of reactive oxygen species that could damage irreplaceable cells [2]. The generation of knockout mice will allow for the determination of the relative contribution of PLAAT1 to cardiolipin content and composition in various tissues.

The generation of *Plaat1*-deficiency models will also allow for the investigation of the role of this enzyme in health and disease. Cardiolipin alterations have been reported in many pathological conditions, and it will be of particular interest to investigate the role of beta-cell PLAAT1 in type 2 diabetes development and prevention given that we, and others [25], have detected this enzyme in the pancreas. Although reactive oxygen species are generated in multiple organelles within dysfunctional beta-cells, the close proximity of cardiolipin to oxidative processes and the enrichment of this lipid with unsaturated fatty acids make it highly susceptible to oxidative damage [9,10,39]. Oxidized cardiolipin can impair the function of the mitochondria and increase proton leak, reducing the quantity of ATP generated in response to incoming glucose, which in turn restricts the insulin-secretory response that results [40]. Oxidized cardiolipin also has a higher binding affinity for cytochrome c, and once externalized to the outer mitochondrial membrane, it is an important pro-apoptotic signaling regulator that can contribute to beta-cell dysfunction and death [14,41]. In this regard, it will be interesting to investigate whether the substrate preference of PLAAT1, which appears to favor the incorporation of saturated fatty acids both in isolated assays and when expressed in cells, will provide some degree of protection from metabolic disorders related to cardiolipin oxidation.

In summary, we have identified PLAAT1 as a fifth enzyme in cardiolipin remodeling, marking the first new discovery in this field in over a decade. PLAAT1 has transacylase activity using MLCL as an acyl acceptor and PC as an acyl donor, with an apparent preference for saturated and monounsaturated fatty acids in the *sn-1* position. Future studies will examine the physiological and pathophysiological roles of this enzyme in vivo.

## 4. Materials and Methods

### 4.1. Animals

All animal procedures were approved by the University of Waterloo Research Ethics Board and Animal Care Committee and were conducted under animal use protocols AUPP#13-13 (approved 28 May 2013) and AUPP#17-18 (approved 27 June 2017). All studies were performed in accordance with the Canadian Council on Animal Care Guidelines. Animals were housed in a temperature- and humidity-controlled environment, on a 12:12 h reversed light/dark cycle, and had ad libitum access to food and water. Male C57BL/6J mice aged 12 weeks were used for studies on endogenous tissue *Plaat1* content and subcellular localization in whole-brain homogenates.

### 4.2. Cell Culture

Human embryonic kidney (HEK)-293 cells from the American Type Cell Culture (ATCC) collection were routinely sub-passaged in 100 mm tissue culture dishes in Dulbecco’s Modified Eagle’s Medium (DMEM) containing 10% fetal bovine serum (FBS) (Sigma Aldrich, Burlington, MA, USA) and 1% penicillin–streptomycin (Gibco, Grand Island, NY, USA).

### 4.3. RNA Extraction, Reverse Transcription (RT), and PCR

Total RNA was isolated from mouse organs using TRIzol reagent and a Polytron homogenizer. cDNA was synthesized from 1 μg of RNA by oligo (dT) priming using a High-Capacity cDNA Reverse Transcription Kit according to the manufacturer’s protocol (Applied Biosystems, Mississauga, ON, Canada). Gene expression of a loading control (*18 s*, forward: 5′-GAT CCA TTG GAG GGC AAG TCA-3′; reverse: 5′-AAC TGC AGC AAC TTT AAT ATA CGC TAT T-3′) or *Plaat1* (transcript variant 1, NM_013751.6, forward: 5′-CTG AGC TGT GAG CAG GCG ATT TGT GTG-3′; reverse; 5′-AGC CAT CAC CCA AGT ACA GTG CCC AG-3′) was analyzed by PCR amplification in a Bio-Rad T100 thermocycler under the following conditions: 95 °C for 4 min, followed by 30 cycles at 95 °C for 30 s, 60 °C for 30 s, 72 °C for 1 min. Samples underwent a final extension at 72 °C for 7 min. The number of cycles was chosen based on a preliminary analysis of the exponential phase range for tissues with the highest expression (data not shown). Amplicons were resolved on a TAE-agarose gel with ethidium bromide for visualization under UV light by a Chemidoc Touch Imaging System, and bands were quantified by densitometric scanning to generate semi-quantitative values (Bio-Rad Laboratories, Mississauga, ON, Canada).

### 4.4. Subcellular Fractionation

Subcellular fractions were separated using homogenates from whole mouse brains as previously described by Dimauro et al. [42]. Subfractionation of the MAM was performed according to the protocol that is described in detail by Wieckowski et al. [43].

### 4.5. Immunoblotting

Protein lysates from subcellular fractions or cells were prepared for immunoblotting in 50 mM Tris-HCl, pH 6.8, 1 mM EDTA, 0.5% Triton X-100. Samples were then mixed with 6X Laemmeli Buffer (125 mM Tris-HCl, pH 6.8, 20% glycerol, 4% SDS, 10% 2-mercaptoethanol, and 0.05% bromophenol blue) and denatured by incubating at 95 °C for 5 min. Samples then underwent gel electrophoresis on 12% SDS-PAGE stain-free gels (Bio-Rad, Mississauga, ON, Canada) at 200 V for 40 min. Total protein loading was visualized by UV imaging of the stain-free gel using a ChemiDoc Touch Imaging System (Bio-Rad), and then proteins were transferred to nitrocellulose membranes at 350 mA for 90 min. Membranes were blocked for 1 h with 5% skim-milk blocker (*w*/*v*) in TBST (50 mM Tris-HCl, pH 7.4, 150 mM NaCl, 0.1% Tween-20) and then probed overnight in TBST with 3% skim-milk blocker (*w*/*v*) using primary antibodies (1:1000 dilution) directed against PLAAT1 (Abnova Taiwan Corporation, Taipei, Taiwan), histone H3 (Cell Signaling, Whitby, ON, Canada), AIF (Cell Signaling), cytochrome c (Cell Signaling), SCD1 (Cell Signaling), and ACSL4 (Sigma Aldrich, Oakville, ON, Canada), or HA (Sigma Aldrich). The following day, samples underwent 3× TBST wash and then were probed with HRP-conjugated antibodies in TBST with 3% skim-milk blocker. Blots were again washed 3× with TBST, and bands were detected by chemiluminescence using a ChemiDoc Touch Imaging System.

### 4.6. Cloning of Ad-Plaat1

The complete coding region of murine *Plaat1*/*Hrasls1* corresponding to transcript variant 1 (NM_013751.6) and encoding for the murine PLAAT1 protein (NP_038779) was amplified by PCR from mouse whole-brain cDNA, and the resulting amplicon was subcloned into pGEM-T-Easy resulting in the production of pGEM-*Plaat1* that was verified by direct sequencing. pGEM-*Plaat1* was used as a template to produce an amplicon containing a C-terminal 6 x histidine tag with NotI/SalI restriction sites that was subcloned into pShuttle-IRES-hrGFP2 NotI/XhoI restriction sites, which was linearized by digestion with PmeI, and transformed into chemically competent BJ-5183 cells pre-transformed with the Ad-1 vector (Agilent Technologies, Santa Clara, CA, USA). Clones containing recombinant adenoviral DNA were verified by PacI digest followed by direct sequencing and were amplified and linearized by PacI digest prior to transfection into HEK-293 cells for formation and amplification of active adenoviral PLAAT1. Control adenovirus was produced by the same method using pShuttle-IRES-hrGFP2 without gene insertion into the multiple cloning site. The amplified virus was titred by serial dilution in cultures of HEK-293 cells grown in 12-well plates, and infectious units (IFU) were quantified by fluorescence microscopy-based counting of infected cells in multiple frames per well.

### 4.7. Gas Chromatography (GC)

Total lipids were extracted for gas chromatography analysis from HEK-293 cells infected for 48 h at 20 IFU/cell using the method of Bligh and Dyer [44]. The organic phase was recovered and dried under a stream of N_2_. Samples were reconstituted in 50 μL of chloroform, applied to a silica gel G plate, and resolved by TLC using a hexane:diethyl ether:glacial acetic acid solvent system (80:20:2, *v*/*v*/*v*) to resolve neutral lipid classes from total phospholipids, which were scraped. To isolate individual phospholipid species, the scraped phospholipid band underwent a double Folch extraction, was dried under N_2_ gas, and was then reconstituted in 50 μL of chloroform. Phospholipids were then resolved by TLC on a silica gel H-plate using a chloroform:methanol:2-propanol:0.25% KCl:trimethylamine solvent front (30:9:25:6:18, *v*/*v*/*v*/*v*/*v*). Bands corresponding to individual phospholipid species were identified using known standards. Identified phospholipids were overlaid with 10 μg of 22:3n-3 ethyl ester internal standard (Nu-Check Prep, Elysian, MN, USA) and scraped for determination of fatty acid composition and content by gas chromatography (GC) with flame ionization detection as previously described [45].

### 4.8. Production of Recombinant Affinity-Purified PLAAT1

HEK-293 cells grown in 150 mm plates were infected at a multiplicity of infection (MOI) of 20 IFU per cell with *Ad-Plaat1* or control adenovirus. Samples were harvested 48 h later; washed with PBS; and lysed in 100 mM Tris-HCl, pH 7.0, 10 mM NaCl, by sonication in an ice-water slurry at 65% output for 3 × 6 s bursts. Unbroken cells and organelles and cellular debris were cleared by centrifugation at 10,000× *g* for 10 min. One hundred microliters of monoclonal anti-HA-antibody bead slurry (Sigma Aldrich, Oakville, ON, Canada) was added to each control and HA-tagged PLAAT1 sample and incubated at 4 °C for 120 min with constant mixing. Samples were then washed in 200 μL PBS. This was repeated 3 times, then beads were reconstituted in 100 μL of PBS. Samples were then ready for the transacylase activity assay. Affinity-purified PLAAT1 was prepared fresh prior to each assay and used in enzymatic reactions immediately after the final wash, without freezing.

### 4.9. PC:MLCL Transacylase Activity of Affinity-Purified PLAAT1

The assay was performed essentially as described by Uyama et al., with minor modifications [3]. Substrate mixtures at 2X final concentrations were prepared, containing 80 μM MLCL and variable concentrations (0–200 μM) of [9,10-^3^H]-18:0/18:0 phosphatidylcholine (distearoyl-PC), [9,10-^3^H]-18:1n-9/18:1n-9 phosphatidylcholine (dioleoyl-PC), or 16:0/[^14^C]-18:2n-6 phosphatidylcholine (1-palmitoyl-2-[^14^C]-linoleoyl-PC), and then mixed and dried under N2 prior to reconstitution by sonification in pre-warmed (37 °C) transacylase reaction buffer (50 mM Tris-HCl, pH 8, 2 mM DTT, 0.1% Nonidet P-40). Reactions were initiated by the addition of 100 μL of pre-warmed substrate mixture to an aliquot of 100 μL of freshly prepared immunoprecipitated PLAAT1–bead complex resulting in a final concentration of MLCL in all reactions of 40 μM. Immunoprecipitate–bead complexes prepared from HEK-293 cells infected only with adenoviral GFP were used as controls in parallel reactions. Reactions were mixed gently and incubated in a 37 °C water bath for 30 min. Reactions were then quenched by the addition of 0.75 mL of methanol:chloroform (2:1, *v*/*v*), and lipids were extracted by the method of Bligh and Dyer. Individual phospholipid species were separated by thin-layer chromatography (Silica Gel Hf, 20 × 20 cm, 250 μM; Analtech Inc., Cole-Parmer Canada, Montreal, QC, Canada) using a chloroform:methanol:2-propanol:0.25% KCl:trimethylamine solvent system (30:9:25:6:18, *v*/*v*/*v*/*v*/*v*). Phospholipid bands were visualized by UV illumination after spraying with 0.1% 2,7-dichlorofluorescein in methanol (*w*/*v*) and identified and scraped based on comparison with cardiolipin standards (Avanti Polar Lipids, Millipore Sigma, Mississauga, ON, Canada). Scraped cardiolipin bands were quantified by liquid scintillation counting in 10 mL of liquid scintillant (Ecolite, MP Biomedicals, Santa Ana, CA, USA). The content of PLAAT1 protein was quantified in samples by immunoblot analysis, where a standard curve was generated on the same blot as the sample using a known quantity of HA-tagged protein (Cat. #HA15-R; Genemed Synthesis Inc, San Antonio, TX, USA). All reactions were run in duplicates, and means represent values from 2–3 separate experiments.

## Figures and Tables

**Table 1:** Kinetic parameters of PLAAT1 with various substrates ^1^.

Substrate	V_max_ (μmol/min/mg protein)	*K_m_* (μM)	V_max_/*K_m_*
[9,10-^3^H]-distearoyl-PC	1.61	126	0.013
[9,10-^3^H]-dioleoyl-PC	0.61	16	0.038
1-palmitoyl-2-[^14^C]-linoleoyl-PC	nd	nd	nd

^1^ Transacylase activity parameters of PLAAT1 were calculated from Lineweaver–Burke plots generated by assaying activity of affinity-purified enzyme using 100 μM MLCL reacted with 0–200 μM PC (*n* = 2–3). nd = not determined.

## Data Availability

Data are available upon reasonable request to the authors.

## Supplementary Materials

The following supporting information can be downloaded at: https://www.mdpi.com/article/10.3390/ijms23126714/s1.
