# Supplementary material for: PLAAT1 Exhibits Phosphatidylcholine:Monolysocardiolipin Transacylase Activity
Source: Int J Mol Sci. 2022 Jun 16;23(12):6714. doi: 10.3390/ijms23126714 (PMC9224490; doi:10.3390/ijms23126714)
Supplement: Supplementary file 1 [file ijms-23-06714-s001.zip › ijms-1728404-supplementary.pdf]

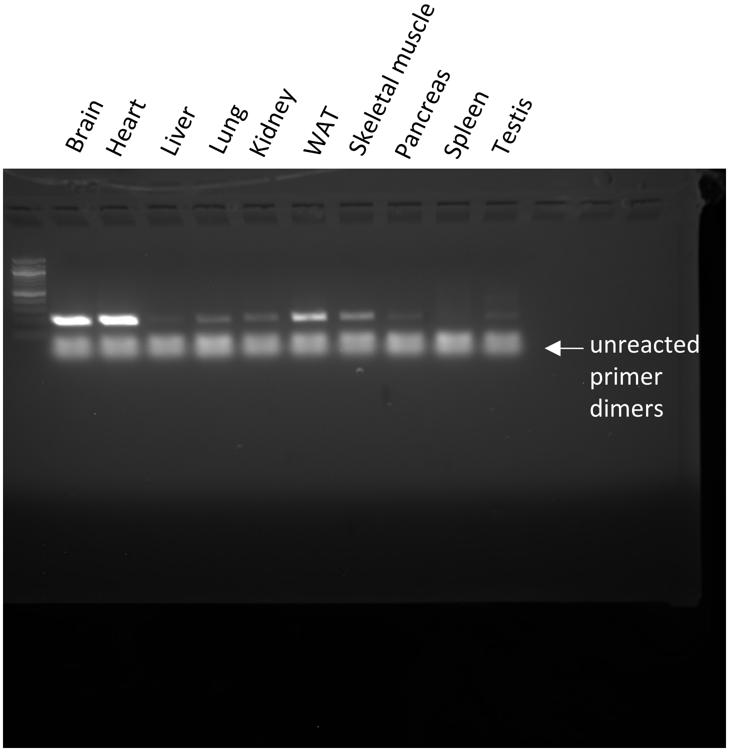

Supplementary Figure S1. RT-PCR image of *Plaat1* expression (uncropped gel image).

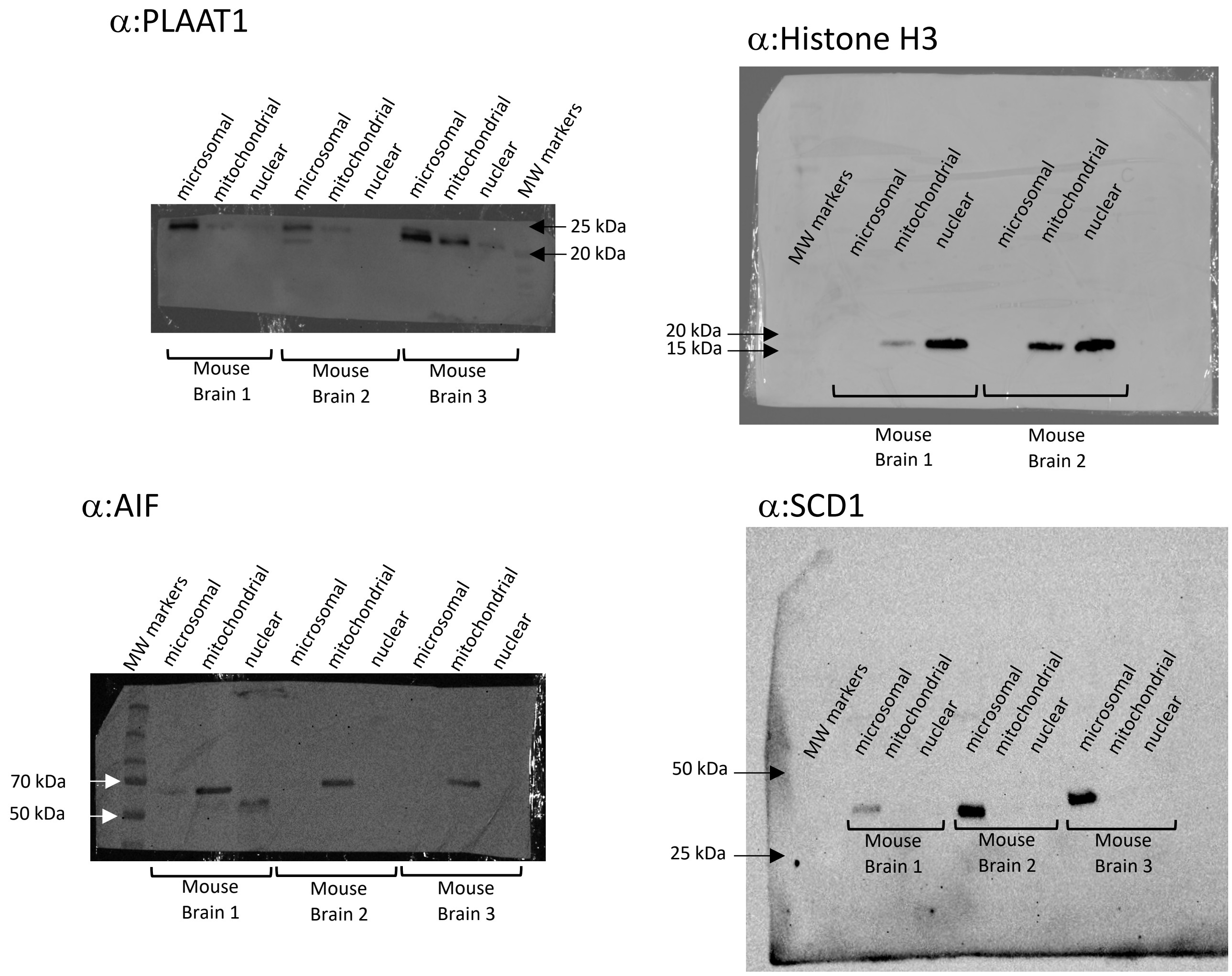

Supplementary Figure S2. Uncropped immunoblots of subcellular fractionation images shown in Figure 1C. Chemiluminescent images are superimposed over photos to show the edge of the blot, and molecular weight (MW) markers for PLAAT1, Histone H3, and AIF images.

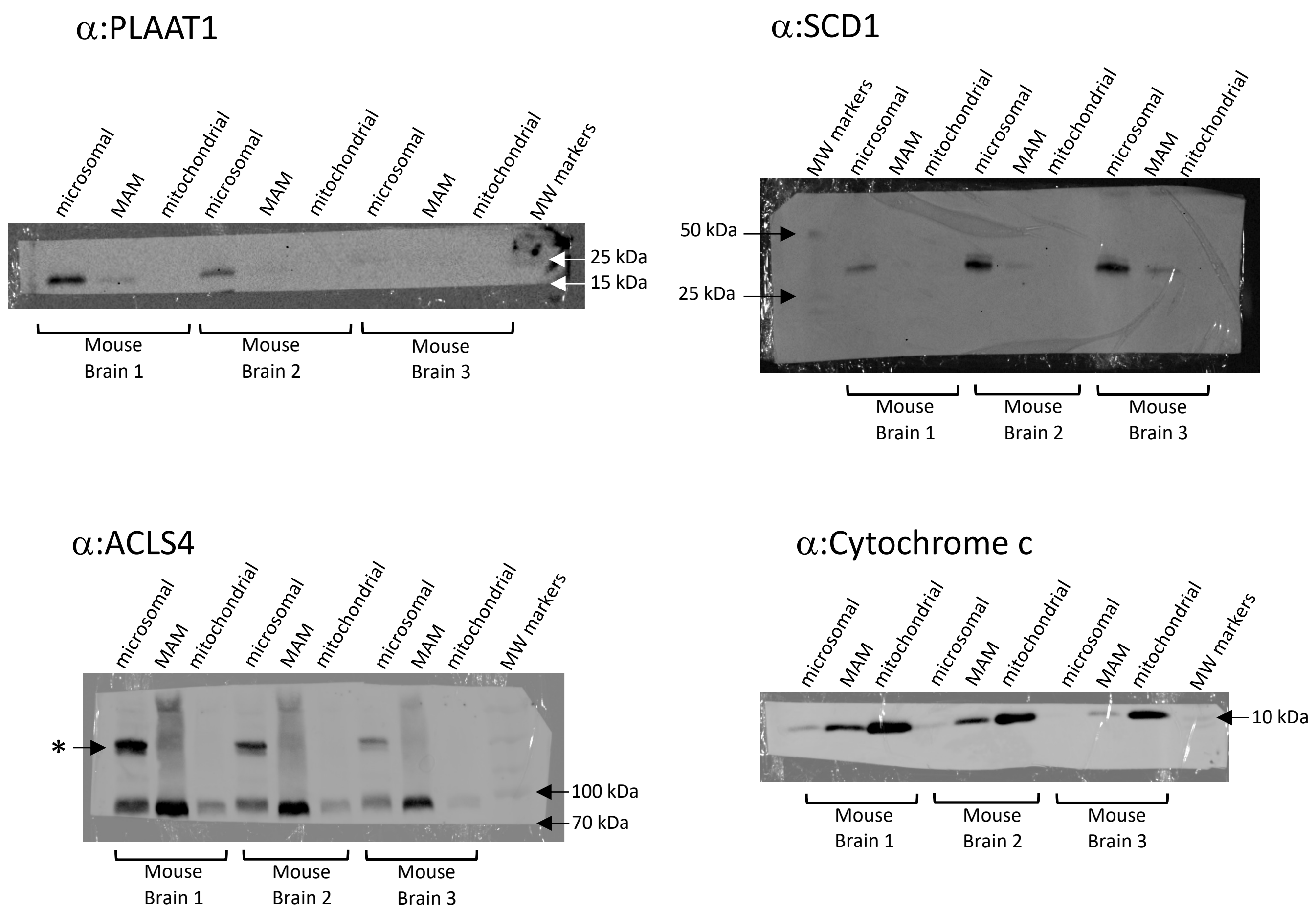

Supplementary Figure S3. Uncropped immunoblots of subcellular fractionation images shown in Figure 1D. Chemiluminescent images are superimposed over photos to show the edge of the blot, and molecular weight (MW) markers on images.
